# Effect of exercising while fasting on eating behaviors and food intake

**DOI:** 10.1186/1550-2783-10-50

**Published:** 2013-11-01

**Authors:** Surabhi Bhutani, Monica C Klempel, Cynthia M Kroeger, Eleanor Aggour, Yolian Calvo, John F Trepanowski, Kristin K Hoddy, Krista A Varady

**Affiliations:** 1 Department of Kinesiology and Nutrition, University of Illinois at Chicago, 1919 West Taylor Street, Room 103, Chicago, IL 60612, USA

## Abstract

**Background:**

Alternate day fasting combined with exercise is effective for weight loss.

**Objective:**

The aim of this study was to examine the behavioral adaptations that occur when ADF is combined with exercise, and to determine how these changes affect weight loss.

**Design:**

Obese subjects (n = 64) were randomized to 1 of 4 groups: 1) combination (ADF + endurance exercise), 2) ADF, 3) exercise, or 4) control, for 12 weeks.

**Results:**

Body weight decreased (P < 0.05) in the combination group (6 ± 4 kg), ADF (3 ± 1 kg), exercise group (1 ± 0 kg), with no change in the control group (0 ± 0 kg). When given the choice, subjects chose to exercise the same amount (*P* = 0.790) on the fast days (48 ± 2%) as feed days (52 ± 2%). Percent of exercise sessions performed on fast day mornings (20 ± 6%) did not differ (P = 0.453) from fast day afternoons (28 ± 5%). Likeliness to cheat on the fast day was not higher if the subject exercised in the afternoon (17 ± 7%) versus the morning (10 ± 5%). Hunger decreased (*P* < 0.05) while satisfaction and fullness increased (*P* < 0.05) post-treatment in the ADF group only. Restrained eating increased (*P* < 0.05) and uncontrolled eating decreased (*P* < 0.05) in the combination and ADF groups.

**Conclusion:**

These findings suggest that endurance exercise is an excellent adjunct therapy to ADF, as it leads to positive behavioral changes that may contribute to long-term steady weight loss.

## Introduction

Alternate day fasting (ADF) is a modified form of calorie restriction comprising a fast day (25% energy intake for 24 h) alternated with a feed day (ad libitum energy intake for 24 h) [1]. Previous reports indicate that ADF is an effective strategy to reduce body weight (5% in 12 weeks) and improve body composition. More recently, it has been shown that combining ADF with exercise leads to greater weight loss (7% in 12 weeks) than what has been seen with ADF or exercise alone [2]. Although these findings are promising, it is still unclear how this combination therapy affects eating behaviors, and how these behavioral changes enhance weight loss. Recent evidence suggests that weight loss in obese individuals is attributed to an increase in cognitive restraint [3-5], reduced disinhibition, lower hunger levels [4,5] and decreased consumption of dietary fat [6]. In view of these findings, key questions that have yet to be addressed in this field include: Are obese individuals able to exercise on the fast day? If so, does exercise increase hunger in a way that causes people to cheat on the fast day? What role does the timing of the exercise session play in determining whether or not the individual will cheat? Does ADF, with or without exercise, elicit positive behavioral changes that may contribute to long-term steady weight loss?

Therefore, the aim of this study was to examine the behavioral adaptations that occur when ADF is combined with endurance training, and to investigate how these changes affect weight loss.

## Materials and methods

### Subjects

As described previously [2], independently living subjects were recruited from the University of Illinois at Chicago campus by means of flyers. Of the 146 interested individuals, 83 were deemed eligible to participate according to a preliminary questionnaire and body mass index (BMI) assessment. Key inclusion criteria were as follows: age 25 to 65 years; BMI between 30 and 39.9 kg/m^2^; weight stable for 3 months prior to the beginning of the study (i.e. less than 5 kg weight loss or weight gain); non-diabetic; no history of cardiovascular disease; lightly active (i.e. <3 h/week of light intensity exercise at 2.5 to 4.0 metabolic equivalents (METs) for 3 months prior to the study); non-smoker; no history of bariatric surgery; and not taking weight loss, lipid or glucose lowering medications. The experimental protocol was approved by the Office for the Protection of Research Subjects at the University of Illinois, Chicago. All volunteers gave informed consent to participate in the trial.

### Experimental design and randomization

A 12-week, randomized, controlled, parallel-arm feeding trial was implemented to test the effects of ADF, exercise, and ADF combined with exercise (combination group) on eating behaviors and weight loss. Eligible subjects were stratified on the basis of BMI, age, and sex, and then randomized into 1 of 4 groups: 1) combination group; 2) ADF group; 3) exercise group; 4) control group.

### Diet protocol

As previously described [2], only the combination and ADF groups participated in the dietary intervention, which consisted of two periods: 1) a 4-week controlled feeding period, and 2) an 8-week self-selected feeding period. During the controlled feeding period (week 1–4) participants consumed 25% of their baseline energy needs on the fast day (24 h) and consumed food ad libitum on each feed day (24 h). Baseline energy requirements were assessed by the Mifflin equation [7]. The diet consisted of a 3-day rotating menu plan, and all fast day meals were prepared in the metabolic kitchen of the Human Nutrition Research Unit (HNRU). Fast day meals were consumed between 12.00 pm and 2.00 pm to ensure that each subject was undergoing the same duration of fasting. Meals were formulated on the basis of the American Heart Association guidelines (30% kcal from fat, 15% kcal from protein, and 55% kcal from carbohydrate). All meals were consumed outside of the research center. Participants were requested to eat only the foods provided on the fast days and to bring back any leftover foods to be weighed and recorded. Calorie-free foods, such as black coffee, tea, and diet sodas were permitted as desired. Subjects were also encouraged to drink plenty of water. During the self-selected feeding period (week 8–12) subjects continued with the ADF regimen but no fast day food was provided to them. Instead, each subject met with a dietician at the beginning of each week to learn how to maintain the ADF regimen at home. Subjects were also taught how to monitor energy intake by reading food labels, reducing portion sizes, and choosing low fat meat and dairy options. Control and exercise group subjects were asked to maintain their regular food habits, and were not provided with any food or dietary counseling.

### Exercise protocol

Only the combination and exercise groups participated the exercise intervention. These subjects participated in a moderate intensity exercise program 3 times per week under supervised conditions, for 12 weeks. Exercise was performed using stationary bikes and elliptical machines at the HNRU. Training intensity was estimated for each individual using an age-predicted heart rate maximum (HRmax) equation [209 − (0.7 × age)] [8] and a Polar Heart Rate Monitor (Polar USA, Inc., NY). At the beginning of the study (weeks 1 to 4), each exercise session ran for 25 min duration and corresponded to 60% of the subject’s HRmax. Training duration and intensity increased incrementally at week 4, 7 and 10 by 5 min and 5% HRmax. As such, by week 10, each subject was exercising for a 40 min duration at an intensity of 75% HRmax. ADF and control subjects were asked to maintain their regular activity habits during the study.

### Weight loss assessment

Body weight was measured weekly to the nearest 0.25 kg in the fasted state using a balance beam scale (HealthOMeter, Sunbeam Products, Boca Raton, FL). Waist circumference was measured by a flexible tape to the nearest 0.1 cm, midway between the lower costal margin and super iliac crest during a period of expiration.

### Adherence to the ADF diet and exercise protocol

During the controlled feeding phase (week 1–4), subjects were instructed to eat only the fast day food provided, and to report any extra food item consumed using an “Extra food log”. The log was collected and reviewed by study personnel each week. If the log indicated that the subject ate an extra food item on a fast day, that day was labeled as “not adherent”. Exercise compliance was assessed by recording attendance at each supervised exercise session. If an exercise session was missed, the subject was required to make up for the missed session that same week.

### Physical activity maintenance assessment

Habitual, free-living physical activity was assessed by a pedometer (Digiwalker SW-200, Yamax Corporation, Tokyo, Japan SW). Subjects wore the pedometer for a 7-d period at week 1 and 12. The pedometer was worn attached to the participant’s waistband during waking hours (except while bathing or swimming), and reset to zero each morning. Number of daily steps were recorded in a pedometer log provided, and the log was collected by study personnel at the weigh-in each week. No subjects were enrolled in an exercise class, and all participants were asked to refrain from joining any exercise programs during the course of the study.

### Eating behavior assessment

A validated visual analog scale (VAS) [9] was used to measure hunger, fullness, and satisfaction with the ADF diet. The scale was completed on each fast day (before bedtime). In brief, the VAS consisted of 100-mm lines, and subjects were asked to make a vertical mark across the line corresponding to their feelings from 0 (not at all) to 100 (extremely) for hunger, satisfaction, or fullness. Quantification was performed by measuring the distance from the left end of the line to the vertical mark. The three-factor eating questionnaire (TFEQ-R18) [10] was used to assess restrained eating (conscious restriction of food intake in order to control body weight), uncontrolled eating (tendency to eat more than usual due to a loss of control over intake), and emotional eating (inability to resist emotional eating cues). The TFEQ consists of 18 items on a 4-point response scale (definitely true/mostly true/mostly false/definitely false). Responses to each of the 18 items are given a score between 1 and 4. The item scores are then summated into scale scores for restrained eating, uncontrolled eating, and emotional eating. The raw scale scores are transformed to a 0–100 scale [((raw score -- lowest possible raw score)/possible raw score range) -- 100]. Higher scores are indicative of greater restrained, uncontrolled, or emotional eating.

### Energy and macronutrient intake

Each participant completed a 3-day food record on 2 feed days during the week, and on 1 feed day during the weekend, at each week of the 12-week trial. Thus, a total of 36 feed day food records were collected for each subject. At baseline, the dietician provided 15 min of instruction to each participant on how to complete the food records. These instructions included verbal information and detailed reference guides on how to estimate portion sizes and record food items in sufficient detail to obtain an accurate estimate of dietary intake. Subjects were instructed to record food items, in as much detail as possible, in the blank food diary provided. Any mixed foods were broken down to individual food items to be recorded one per line. Food records were collected at the weigh-in each week, and were reviewed by the dietician for accuracy and completeness. All dietary information from the food records was entered into the food analysis program, Nutritionist Pro (Axxya Systems). The program was used to calculate the total daily intake of energy, protein, carbohydrate, fat, cholesterol, and fiber.

### Statistical analysis

Results are presented as mean ± SEM. Differences between intervention groups at baseline were analyzed by a one-way ANOVA. Within-group differences were analyzed using repeated-measures ANOVA. An intention-to-treat analysis was performed for all variables measured. A P value of < 0.05 was used as a criterion for statistical significance in all analyses. Data were analyzed using SPSS software (version 20.0 for Mac OSX; SPSS Inc, Chicago, IL, USA).

## Results

### Subject baseline characteristics and weight loss

As reported previously [2], 83 subjects began the clinical trial (combination: n = 18, ADF: n = 25, exercise: n = 24, control: n = 16), and sixteen subjects finished in each intervention group (total n = 64). Additional subjects were randomized to groups with high dropout rates, such as the ADF and exercise group, to ensure that the number of subjects would be the same in each group at the end of the trial. Dropouts were primarily due to scheduling conflicts. Baseline characteristics are reported in Table 1. There were no between-group differences for age, sex, ethnicity, body weight, height, BMI, or waist circumference. Body weight decreased (P < 0.05) in the combination group (6 ± 4 kg), ADF (3 ± 1 kg) and exercise group (1 ± 0 kg), with no change in the control group (0 ± 0 kg). The decrease in waist circumference was greater (P < 0.001) in the combination group (8 ± 1 cm) compared to the ADF (5 ± 1 cm), exercise group (3 ± 1 cm), and control group (1 ± 1 cm).

**Table 1:** Subject characteristics at baseline

	**Combination**	**ADF**	**Exercise**	**Control**	**P-value**^**1**^
n	18	25	24	16	
Age (y)	45 ± 5	42 ± 2	42 ± 2	49 ± 2	0.158
Sex (F/M)	18 / 0	24 / 1	23 / 1	15 / 1	0.266
Ethnicity (n)					
African American	7	12	11	11	
Caucasian	5	7	6	3	
Hispanic	6	6	4	2	
Other	0	0	3	0	
Body weight (kg)	91 ± 6	94 ± 3	93 ± 2	93 ± 5	0.904
Height (cm)	160 ± 0	163 ± 0	162 ± 0	162 ± 1	0.896
BMI (kg/m^2^)	35 ± 1	35 ± 1	35 ± 1	35 ± 1	0.934
Waist circumference	96 ± 2	100 ± 2	98 ± 2	99 ± 3	0.636

Values reported as mean ± SEM. Intention to treat analysis. BMI: Body mass index, F: Female, M: Male.

^1^P-value between groups at baseline: One-way ANOVA.

### ADF and exercise compliance

The combination group attended 95 ± 2% of the exercise sessions while the exercise group attended 94 ± 1% of the sessions. There was no difference (P = 0.83) in exercise compliance between groups. Adherence to the fast day diet remained high in the combination (81 ± 7%) and ADF group (80 ± 9%) throughout the course of the trial. No between-group differences were observed in fast day diet adherence when the combination group was compared to the ADF group (P = 0.23). As for regular physical activity, there were no differences in steps/d between groups or within groups from baseline to post-treatment: combination (week 1: 5566 ± 656, week 12: 6018 ± 765), ADF (week 1: 4031 ± 752, week 12: 4920 ± 664), exercise (week 1: 5381 ± 885, week 12: 5998 ± 767), and control group (week 1: 6458 ± 749, week 12: 6206 ± 736).

### Timing of the fast day exercise session and impact on food intake

**Figure 1:**
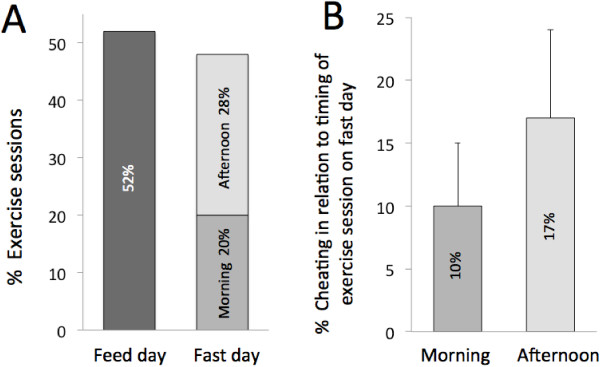
**Timing of the fast day exercise session and impact on food intake. A**. Percent of exercise sessions scheduled by subjects on feed days versus fast days (morning and afternoon). **B**. Percent of cheating on the fast day (i.e. eating more than the prescribed amount of energy) in relation to timing of the exercise session. No difference in cheating in relation to exercising in the morning versus the afternoon on the fast day (One-way ANOVA).

Subjects were given the option of scheduling their exercise sessions on feed days or fast days (morning or afternoon). Figure 1A portrays the percent of exercise sessions held on feed versus fast days. Combination group subjects showed no preference (*P* = 0.790) towards exercising on feed days (52 ± 2%) versus fast days (48 ± 2%). Furthermore, percent of exercise sessions performed on fast day mornings (20 ± 6%) did not differ (P = 0.453) from those performed on fast day afternoons (28 ± 5%). We also wanted to determine if subjects cheated more on the fast day (i.e. ate more than their prescribed amount of energy) if they exercised in the morning versus the afternoon. Results reveal that likeliness to cheat was not significantly higher if the subject chose to exercise in the afternoon (17 ± 7%) versus the morning (10 ± 5%) (Figure 1B).

### Changes in eating behaviors

Changes in fast day eating behaviors are presented in Table 2. No change in hunger, satisfaction or fullness was observed in the combination group post-treatment. However in ADF subjects, hunger decreased (*P* < 0.05) while satisfaction and fullness increased (*P* < 0.05) after 12 weeks of treatment. Restrained eating increased (*P* < 0.05) by 16 ± 6 and 10 ± 2 in the combination and ADF group, respectively, after 12 weeks. Uncontrolled eating decreased (*P* < 0.05) in the combination (14 ± 4) and ADF group (6 ± 3) over the course of the trial. Emotional eating decreased (*P* < 0.01) only in the combination group (15 ± 5). No changes were observed in eating behaviors in the exercise and control group.

**Table 2:** Changes in eating behaviors during the 12-week study

	**Intervention**	**Week 1**	**Week 12**	**P-value**^**1**^	**P-value**^**2**^	**Change**^**3**^	**P-value**^**4**^
Hunger (mm)	Combination	5.7 ± 0.5	4.7 ± 0.7	0.185	0.941	−1.0 ± 0.7	0.495
	ADF	6.3 ± 0.5	4.7 ± 0.7	0.034		−1.6 ± 0.7	
Satisfaction (mm)	Combination	3.8 ± 0.8	4.1 ± 0.6	0.575	0.817	0.3 ± 0.5	0.240
	ADF	3.2 ± 0.4	4.3 ± 0.6	0.031		1.1 ± 0.5	
Fullness (mm)	Combination	3.7 ± 0.8	4.0 ± 0.7	0.564	0.967	0.3 ± 0.5	0.146
	ADF	2.4 ± 0.4	4.0 ± 0.7	0.016		1.6 ± 0.6	
Restrained eating score	Combination	40 ± 4	56 ± 7	0.029	0.207	16 ± 6 ^a^	0.013
	ADF	52 ± 2	62 ± 3	0.006		10 ± 2 ^a^	
	Exercise	49 ± 3	49 ± 3	0.944		0 ± 2 ^b^	
	Control	47 ± 7	48 ± 6	0.828		1 ± 6 ^a,b^	
Uncontrolled eating score	Combination	44 ± 3	30 ± 4	0.007	0.050	−14 ± 4 ^a^	0.002
	ADF	35 ± 3	29 ± 3	0.023		−6 ± 3 ^a,b^	
	Exercise	40 ± 4	39 ± 5	0.783		−1 ± 2 ^b^	
	Control	23 ± 4	28 ± 6	0.152		5 ± 3 ^b^	
Emotional eating score	Combination	57 ± 5	42 ± 6	0.002	0.063	−15 ± 5 ^a^	0.005
	ADF	38 ± 5	35 ± 5	0.428		−3 ± 3 ^b^	
	Exercise	58 ± 7	56 ± 7	0.447		−2 ± 3 ^b^	
	Control	38 ± 12	38 ± 11	0.584		0 ± 5 ^b^	

Values reported as mean ± SEM. Intention to treat analysis. ADF: Alternate day fasting.

^1^P-value between week 1 and week 12: Repeated-measures ANOVA.

^2^P-value between groups at week 12: One-way ANOVA.

^3^Absolute change between week 1 and 12 values.

^4^P-value between groups for absolute change: One-way ANOVA. Means not sharing a common superscript letter are significantly different (Tukey post-hoc test).

### Impact of the fast day exercise session on eating behaviors

Hunger did not increase if the subject exercised on a fast day (week 1: 6 ± 1, week 12: 4 ± 2, P = 0.240). Fullness did not decrease if the subject exercised on a fast day (week 1: 4 ± 2, week 12: 4 ± 1, P = 0.653). Moreover, satisfaction with the ADF diet did not decrease if the subject exercised on a fast day (week 1: 4 ± 2, week 12: 4 ± 1, P = 0.549).

### Changes in energy and macronutrient intake on feed days

Dietary intake for each intervention group is reported in Table 3. No changes were observed for energy, protein, carbohydrate, fat, cholesterol, and fiber after 12 weeks of treatment.

**Table 3:** Energy and macronutrient intake on feed days during the 12-week study

	**Intervention**	**Week 1**	**Week 12**	**P-value**^**1**^	**P-value**^**2**^	**Change**^**3**^	**P-value**^**4**^
Energy (kcal)	Combination	1656 ± 265	1358 ± 123	0.278	0.854	−298 ± 260	0.897
	ADF	1681 ± 155	1457 ± 204	0.228		−224 ± 173	
	Exercise	1623 ± 145	1553 ± 135	0.739		−70 ± 203	
	Control	1607 ± 307	1416 ± 207	0.360		191 ± 190	
Protein (g)	Combination	70 ± 21	63 ± 14	0.903	0.958	−7 ± 23	0.581
	ADF	65 ± 10	70 ± 10	0.115		5 ±10	
	Exercise	60 ± 5	62 ± 8	0.467		−2 ± 8	
	Control	71 ± 9	68 ± 5	0.817		3 ± 12	
Carbohydrate (g)	Combination	199 ± 35	164 ± 19	0.547	0.801	−35 ± 38	0.928
	ADF	200 ± 19	161 ± 19	0.155		−39 ± 24	
	Exercise	202 ± 25	177 ± 20	0.470		−25 ± 33	
	Control	182 ± 34	140 ± 31	0.21		−42 ± 28	
Fat (g)	Combination	64 ± 10	50 ± 7	0.454	0.793	−14 ± 11	0.983
	ADF	69 ± 8	59 ± 13	0.327		−10 ± 9	
	Exercise	64 ± 11	66 ± 6	0.717		2 ± 13	
	Control	66 ± 16	65 ± 11	0.780		−1 ± 12	
Saturated fat (g)	Combination	23 ± 3	19 ± 2	0.412	0.599	−4 ± 3	0.815
	ADF	28 ± 2	26 ± 5	0.831		−2 ± 4	
	Exercise	23 ± 3	28 ± 3	0.700		5 ± 5	
	Control	27 ± 7	26 ± 4	0.682		−1 ± 5	
Monounsaturated fat (g)	Combination	25 ± 3	20 ± 3	0.375	0.975	−5 ± 4	0.716
	ADF	24 ± 3	21 ± 6	0.969		−3 ± 5	
	Exercise	24 ± 4	22 ± 2	0.118		−2 ± 3	
	Control	23 ± 5	24 ± 4	0.915		1 ± 5	
Polyunsaturated fat (g)	Combination	16 ± 2	11 ± 2	0.309	0.725	−5 ± 3	0.930
	ADF	17 ± 2	12 ± 2	0.452		−5 ± 3	
	Exercise	17 ± 3	16 ± 2	0.294		−1 ± 3	
	Control	16 ± 3	15 ± 3	0.926		−1 ± 4	
Fiber (g)	Combination	18 ± 3	16 ± 2	0.609	0.280	−2 ± 4	0.657
	ADF	16 ± 2	11 ± 2	0.078		−5 ± 2	
	Exercise	18 ± 2	12 ± 2	0.036		−6 ± 3	
	Control	11 ± 3	10 ± 2	0.832		−1 ± 5	
Cholesterol (mg)	Combination	245 ± 34	268 ± 47	0.744	0.868	23 ± 43	0.391
	ADF	329 ± 83	225 ± 58	0.225		−104 ± 79	
	Exercise	223 ± 49	227 ± 53	0.955		4 ± 69	
	Control	380 ± 73	272 ± 25	0.120		−108 ± 57	

Values reported as mean ± SEM. Intention to treat analysis. ADF: Alternate day fasting.

^1^P-value between week 1 and week 12: Repeated-measures ANOVA.

^2^P-value between groups at week 12: One-way ANOVA.

^3^Percent change between week 1 and week 12 values.

^4^P-value between groups for percent change: One-way ANOVA. Means not sharing a common superscript letter are significantly different (Tukey post-hoc test).

## Discussion

Our findings show, for the first time, that endurance exercise can be easily incorporated into the ADF regimen. Specifically, subjects were able to exercise on the fast day, and this extra energy expenditure did not translate into increased hunger or extra food intake. We also show here that ADF combined with exercise improves several eating behaviors. For instance, after 12 weeks of treatment, restrained eating was increased while uncontrolled eating and emotional eating were decreased in obese individuals.

Our primary goal in this study was to see if subjects undergoing ADF can exercise on the fast day. All subjects exercised 3 times per week under supervised conditions. Throughout the study, subjects were allowed to choose the day (feed or fast day) and time (morning: 7.00-11.00 am, or afternoon: 3.00-7.00 pm) of the exercise sessions. Results reveal that the combination group showed no preference towards exercising on feed days (52%) versus fast days (48%). Moreover, the percent of exercise sessions performed on fast day mornings (20%) did not differ from those performed on fast day afternoons (28%). We also wanted to see if the negative energy balance produced by the physical activity would lead to higher energy intake on the fast day. We hypothesized that the subjects exercising on fast day afternoons would be more likely to cheat (i.e. surpass their prescribed fast day energy goal) compared to subjects exercising in the morning. We assumed that cheating would be higher in the afternoon exercisers, as hunger peaks 30–40 minutes post workout [11]. Since the morning exercisers would be able to eat their fast day meal shortly after their exercise session (12.00-2.00 pm), they would be satisfied and less likely to cheat. In contrast, the afternoon exercisers would not have another meal to eat after their exercise session, which may lead them to consume extra food to suppress the post-workout hunger. Interestingly, the likeliness to cheat was not significantly higher in the afternoon exercisers (17%) compared to the morning exercisers (10%). However, it is possible that this difference was not significant due to small sample size (n = 16). Similar to our trial, Maraki et al. studied the acute effect of one hour of morning (post breakfast) and afternoon (pre dinner) exercise on hunger and energy intake [12]. Both morning and afternoon exercisers experienced increases in hunger, but did not exhibit increased energy intake. Our findings parallel those of Maraki et al. [12] in that we also saw no increase in energy intake post-workout.

The effect of ADF with or without exercise on hunger, satisfaction and fullness was also tested. After 12 weeks of treatment, hunger decreased while satisfaction and fullness increased in the ADF group. The effect of ADF on eating behaviors was also tested by Heilbronn et al. Normal weight subjects participated in an ADF regimen (100% calorie restriction on the fast day) for 3 weeks. After this short intervention period, fullness increased, but there were no changes in the perception of hunger or satisfaction [13]. The findings of Heilbronn et al. may have differed from ours because their study employed a true ADF regimen (complete fast on the fast day) whereas we used a modified ADF regimen (75% restriction on the fast day). Since the Heilbronn et al. subjects were not allowed to eat anything on the fast day, this may explain why hunger remained elevated throughout the course of the trial. In contrast to the ADF group, the combination group did not demonstrate any changes in hunger, satisfaction or fullness in the current study. The reason for this is not clear. Blundell et al. [14] investigated the effect of medium term (7–16 d) moderate intensity endurance exercise on appetite stimulation. Findings reveal that hunger and food intake increased post-exercise in order to compensate for the negative energy balance achieved with training [14]. In contrast, Guelfi et al. demonstrated that 12 weeks of 40–60 minutes of moderate intensity exercise (70–80% HRmax) produced opposite results [15]. Specifically, Guelfi et al. showed no change in perceived hunger, while levels of perceived fullness increased [15]. It should be noted however, that subjects in the Blundell et al. [14] study were required to expend approximately 1000 kcal/d with exercise. This level of energy expenditure is far greater than that of our study (estimated to be 150–250 kcal/d). Thus, increases in hunger post-exercise may only occur if energy expenditure with exercise meets or exceeds 1000 kcal/d. Nevertheless, in light of these contradictory findings, the impact of combination diet and exercise therapies on hunger and fullness warrant further investigation.

Changes in restrained eating, uncontrolled eating, and emotional eating were also examined. In both the ADF and combination groups, restrained eating increased while uncontrolled eating decreased. These positive changes in eating behaviors are most likely due to the subjects’ involvement in weekly dietary counseling [16]. As for emotional eating, only the combination group experienced decreases in this parameter. It is possible that emotional eating was not decreased in the ADF group due to the lack of the exercise intervention. Positive changes in mood have been previously reported with short bouts of exercise [12,17]. Pendleton et al. designed a trial to study the effect of cognitive behavior therapy with or without exercise on binge eating in obese women. After 16 months, only the group that was exercising experienced improvements in mood, which resulted in decreased binge eating [18]. Taken together, it is possible that the combination of ADF plus exercise may have better overall effects on these eating behaviors than each intervention alone.

We also wanted to examine the ability of our dietary counseling program to aid individuals in reducing energy intake. Subjects met with a dietician each week to learn how to ascertain the caloric content of foods, control portion sizes, read food labels, and avoid high fat foods. Dietary intake was measured using a 3-day food record that was completed each week (on feed days). After 12 weeks of treatment, energy intake decreased by approximately 300 kcal in the combination group and by 220 kcal in ADF group, though not significantly. These reported energy deficits are somewhat lower than expected given that the combination and ADF group lost 7 kg and 3 kg, respectively. These incongruences between weight loss and energy deficits are most likely due to reporting errors in the food records. In view of this, more robust measures to assess energy expenditure and intake, such as the doubly labeled water technique, should be used in future trials of ADF.

This study has several limitations. First, the effect of exercise alone on hunger, fullness and satisfaction levels was not measured in this study. This makes it impossible to tease apart the effects of ADF versus exercise on these parameters. Secondly, the sample size employed (n = 16 per group) may have been too small to detect differences between groups for certain variables such as energy intake, and likeliness to cheat post-exercise. Thirdly, we implemented food records to measure energy intake, when we should have used a more accurate method, such as the doubly labeled water technique.

In summary, our results suggest that an endurance exercise program can be easily incorporated into the ADF regimen. Adding exercise to ADF does not increase the likeness to cheat on the fast day, which ensures that weight loss will be sizeable and consistent. We also show that the combination of ADF plus exercise increases restrained eating while decreasing uncontrolled and emotional eating. Taken together, endurance exercise is an excellent adjunct therapy to ADF, as it leads to positive behavioral changes that may contribute to long-term steady weight loss.

## Competing interests

The authors have no competing of interest to report.

## Authors’ contributions

SB designed the experiment, conducted the clinical trial, analyzed the data, and wrote the manuscript. MCK and CMK assisted with the conduction of the clinical trial. EA, YC, JFT, KKH assisted with the data analysis. KAV assisted with the design of the experiment, and wrote the manuscript. All authors read and approved the final manuscript.
